# Comparing the effectiveness of various cumulative doses of cisplatin in treating locally advanced nasopharyngeal cancer during the period of intensity-modulated radiotherapy: a meta-analysis

**DOI:** 10.3389/fmed.2026.1829699

**Published:** 2026-06-03

**Authors:** Tao Lin, Keyi Lin, Yunqiu Zheng

**Affiliations:** 1 Department of Otolaryngology, The Third People’s Hospital of Yibin, Yibin, Sichuan, China; 2 Jiangxi Medical College, Nanchang University, Nanchang, Jiangxi, China; 3 Gaoxian Center for Disease Control and Prevention, Yibin, Sichuan, China

**Keywords:** cisplatin, cumulative dose, distant metastasis-free survival, intensity-modulated radiotherapy, meta-analysis, nasopharyngeal carcinoma

## Abstract

**Objective:**

In the age of intensity-modulated radiotherapy (IMRT), the best cumulative cisplatin dose (CCD) for concurrent chemoradiotherapy for locally advanced nasopharyngeal cancer (LA-NPC) is still up for debate. In order to guide clinical dose decisions, this meta-analysis sought to examine the effects of various CCD on survival outcomes.

**Methods:**

A thorough search was conducted in PubMed, Embase, Web of Science, and Cochrane Library through December 2025. Randomized controlled trials or observational studies comparing CCD thresholds (≥200 mg/m^2^ vs. <200 mg/m^2^) in IMRT-treated patients were included. Overall survival (OS) and progression-free survival (PFS) were the main results. Treatment-related toxicities and distant metastasis-free survival (DMFS) were secondary outcomes. Random-effects models were used to pool hazard ratios (HRs) with 95% confidence intervals (CIs) for survival outcomes. Relative risks (RRs) with 95% CIs were computed for toxicities (≥2 trials). To investigate the robustness of the results, subgroup and sensitivity analyses were carried out.

**Results:**

Included were 10 retrospective cohort studies with 11,220 individuals. OS displayed a favorable trend approaching statistical significance (HR = 0.65, 95% CI: 0.51–0.82). Moderate heterogeneity was observed (I^2^ = 50.4%, *P* for heterogeneity = 0.060). PFS showed no significant difference between dose groups (HR = 0.81, 95% CI: 0.54–1.22), with substantial heterogeneity (I^2^ = 81.0%, *P* for heterogeneity <0.001). Higher CCD was associated with significantly improved DMFS (HR = 0.54, 95% CI: 0.40–0.74), with moderate heterogeneity (I^2^ = 61.4%, *P* for heterogeneity = 0.011). Subgroup analysis revealed that the OS and DMFS benefit was consistent across treatment modalities, while the PFS effect differed significantly by treatment modality (*p* < 0.001). For toxicity, the lower-dose group demonstrated significantly reduced risks of any-grade leukopenia (RR = 0.91, 95% CI: 0.86–0.97, *p* = 0.002), any-grade ALT elevation (RR = 0.69, 95% CI: 0.58–0.82, *p* < 0.001), any-grade creatinine elevation (RR = 0.73, 95% CI: 0.60–0.88, *p* = 0.001), any-grade hearing impairment (RR = 0.81, 95% CI: 0.70–0.94, *p* = 0.005), and any-grade skin fibrosis (RR = 0.77, 95% CI: 0.64–0.92, *p* = 0.004), suggesting a more favorable safety profile.

**Conclusion:**

Higher CCD improves DMFS in LA-NPC patients with IMRT, with a near-significant OS trend. However, this survival benefit must be balanced against increased risks of certain acute and late toxicities associated with higher doses. These findings support risk-adapted dose intensification strategies while emphasizing individualized decision-making that integrates efficacy, toxicity, and patient characteristics.

## 1 Introduction

A malignant tumor that arises from the nasopharynx’s mucosal epithelium, nasopharyngeal carcinoma (NPC) has distinct epidemiological features and is very common in southern China and Southeast Asia (1). Concurrent chemoradiotherapy (CCRT) based on cisplatin is presently the standard treatment for locally advanced-NPC (LA-NPC). However, the optimal cumulative dose of cisplatin in this regimen remains a core clinical controversy (2). Theoretically, a higher cumulative dose may offer survival benefits, but it is also bound to be accompanied by more severe toxicities such as nausea and vomiting, renal injury, and bone marrow suppression (3, 4). These toxicities not only affect the quality of life of patients but also often lead to dose reduction or treatment interruption, potentially offsetting the therapeutic advantages and making it difficult for clinicians to balance efficacy and toxicity.

The widespread use of intensity-modulated radiotherapy (IMRT) in the treatment of nasopharyngeal carcinoma has led to a significant improvement in patient survival outcomes in recent years (5). This technological advancement is not only reflected in the increase in survival rates but also in the protection of normal tissues. Compared with previous techniques, IMRT can increase the dose to the target area while significantly reducing the radiation dose to critical organs such as the parotid glands and temporal lobes, thereby effectively reducing long-term complications such as xerostomia and radiation-induced brain injury (6, 7). Given that the toxicity of chemoradiotherapy is directly related to the exposure of normal tissues, the protective effect brought by IMRT is expected to reduce treatment-related acute toxicities (8). This change may reshape the toxicity profile of CCRT, especially the pattern and severity of cisplatin-related toxicities, and potentially affect the clinical feasibility of high-dose cisplatin regimens. Therefore, in the context where IMRT has become the mainstream radiotherapy technique and has improved overall survival (OS), it is clinically necessary to re-examine the relationship between the cumulative dose of cisplatin and the balance between efficacy and toxicity.

Current research conclusions on this issue are inconsistent, and most studies were conducted before the widespread use of IMRT or mixed different radiotherapy techniques, with limited guidance value for current clinical practice (9). To provide an updated synthesis of evidence from the IMRT era, we conducted a systematic review and meta-analysis to compare survival outcomes and adverse events between a cumulative cisplatin dose of ≤200 mg/m^2^ and >200 mg/m^2^ in patients with locally advanced nasopharyngeal carcinoma treated with IMRT.

## 2 Materials and methods

### 2.1 Search strategy

The Preferred Reporting Items for Systematic Reviews and Meta-Analyses (PRISMA) guideline was followed in conducting this systematic review and meta-analysis. Two independent reviewers systematically searched PubMed, Embase, the Cochrane Library, and Web of Science for relevant literature published from database inception to December 31, 2025. The search utilized keywords including: “nasopharyngeal carcinoma,” “Nasopharynx Cancers,” “Cancer,” “Nasopharynx,” “NPC,” “cisplatin,” “cis-Platinum,” “cumulative dose.” For deduplication, all recovered records were loaded into the Zotero reference management program. After deduplication, all unique records’ titles and abstracts were separately evaluated by two reviewers using the predetermined eligibility criteria. After that, the complete texts of research that might qualify were evaluated separately. Any disagreements were settled by conversation or by consulting a third reviewer. To find any further publications that qualified, the reference lists of all included research and pertinent review articles were also manually screened.

### 2.2 Inclusion criteria

The inclusion criteria were developed using the Population, Intervention, Comparison, Outcomes, and Study design (PICOS) framework. (1) Participants: patients with pathologically confirmed, non-metastatic locally advanced nasopharyngeal carcinoma according to the AJCC/UICC staging system (7th edition: stage II–IVb; 8th edition: stage II-IVA; all M0). All patients must have been treated in the IMRT era, with IMRT used as the primary radiotherapy technique for the majority of patients. (2) Intervention: patients who received a higher cumulative dose of cisplatin during concurrent chemoradiotherapy; (3) Comparison: patients who received a lower cumulative dose of cisplatin during the same treatment regimen. The specific dose threshold was adopted as defined in the original studies. (4) Outcomes: OS and progression-free survival (PFS) were the main results. One of the secondary outcomes was distant metastasis-free survival (DMFS). For at least one of the survival outcomes, studies had to give hazard ratios (HRs) with 95% confidence intervals (CIs) or supply the data needed to calculate them. For toxicity outcomes, incidence rates of grade 3–5 adverse events (AEs) were extracted. When information from at least two trials was available for a particular toxicity class, relative risks (RRs) with 95% CIs were computed. (5) Research design: Both observational studies and randomized controlled trials (RCTs) might be included.

### 2.3 Exclusion criteria

Research that satisfied any of the following requirements was eliminated: Patients with metastatic (M1) or recurrent nasopharyngeal cancer were included. (2) The study period was predominantly outside the IMRT era, or IMRT was used in less than 80% of the study population. (3) Focused on treatment regimens where non-cisplatin chemotherapies were dominant or the study did not provide a cisplatin dose-stratified analysis. (4) Were reviews, editorials, case reports, conference abstracts, or other non-original research articles. (5) Had a sample size of fewer than 30 patients in the dose-comparison analysis. (6) Were not published in English. (7) Were single-arm studies or studies lacking an internal control group.

### 2.4 Study selection and data extraction

The study selection was carried out separately by two reviewers. Articles that were obviously unrelated were eliminated from the initial screening, which was based on the titles and abstracts of all retrieved entries. For records that appeared eligible or where relevance was uncertain, the full-text publications were obtained and thoroughly assessed by both reviewers. The PRISMA flowchart displays the grounds for exclusion that were noted during the full-text stage. At any point throughout the selection process, disagreements were settled by discussion or by seeking advice from a third senior researcher.

The two reviewers used a pre-made form to separately extract the data. A third reviewer was consulted or a conversation was held to settle any disputes over the retrieved data. First author, publication year, country, study design, total sample size, CCD ≤ 200 mg/m^2^ group sample size, CCD > 200 mg/m^2^ group sample size, age, sex distribution, tumor stage, treatment modality, IMRT confirmation rate, median follow-up duration, outcome measures, and incidence of grade 3–5 adverse events were among the data that were extracted. Hazard ratios with 95% confidence intervals for overall survival, progression-free survival, and distant metastasis-free survival were included in the outcome data.

When multiple HR estimates were available, adjusted HRs from multivariate Cox models were preferentially extracted. For studies that reported HRs based on different comparator definitions, for example CCD > 200 mg/m^2^ vs. CCD = 0 mg/m^2^, the low-dose group was redefined as CCD ≤ 200 mg/m^2^ by combining the relevant subgroups to align with the pre-specified dose threshold. For studies that did not directly report the required HR, estimates were reconstructed from Kaplan–Meier curves using Engauge Digitizer version 12.1. Cohort design differences such as the use of propensity score matching were not used as exclusion criteria; their potential impact was assessed through sensitivity analyses restricted to high-quality studies with NOS scores ≥8.

### 2.5 Assessment of risk of bias

Two reviewers used design-specific methods to independently assess the risk of bias in the included studies. The Newcastle-Ottawa Scale (NOS) (10) was used to evaluate observational research, which assesses the quality of studies using three criteria: result, comparability, and selection. A star-based scoring system with a maximum of nine stars was used. A study was deemed to have high methodological quality if its NOS score was ≥7. A third senior reviewer was consulted or a discussion was used to settle any disputes between the two reviewers.

### 2.6 Statistical analysis

R software version 4.5.2 (R Foundation for Statistical Computing, Vienna, Austria) was used for all statistical analyses. Pooled estimates were presented as hazard ratios with matching 95% confidence intervals for time-to-event outcomes. When HRs were not directly reported, digitizing software (Engauge Digitizer, version 12.1) was used to extract numerical data from Kaplan–Meier survival curves, and the techniques outlined by Tierney et al. were used to estimate HRs with variances. Pooling was done using the inverse variance method. The Cochran’s *Q* test was used to evaluate heterogeneity across trials, and the I2 statistic was used to quantify it. Initially, a fixed-effect model was used; however, a random-effects model was used if considerable heterogeneity was found (defined as *p* < 0.10 for the *Q* test or I^2^ > 50%). Based on induction chemotherapy use, pre-specified subgroup analyses were carried out to investigate possible sources of heterogeneity. To assess the robustness of the pooled data, sensitivity analyses were carried out by gradually eliminating individual research. Funnel plots were used to visually evaluate publication bias. When 10 or more studies were available for a certain outcome, Egger’s regression test was designed. A *p*-value of less than 0.05 was regarded as statistically significant, and all tests were two-sided.

## 3 Results

### 3.1 Literature screening process

**Figure 1:**
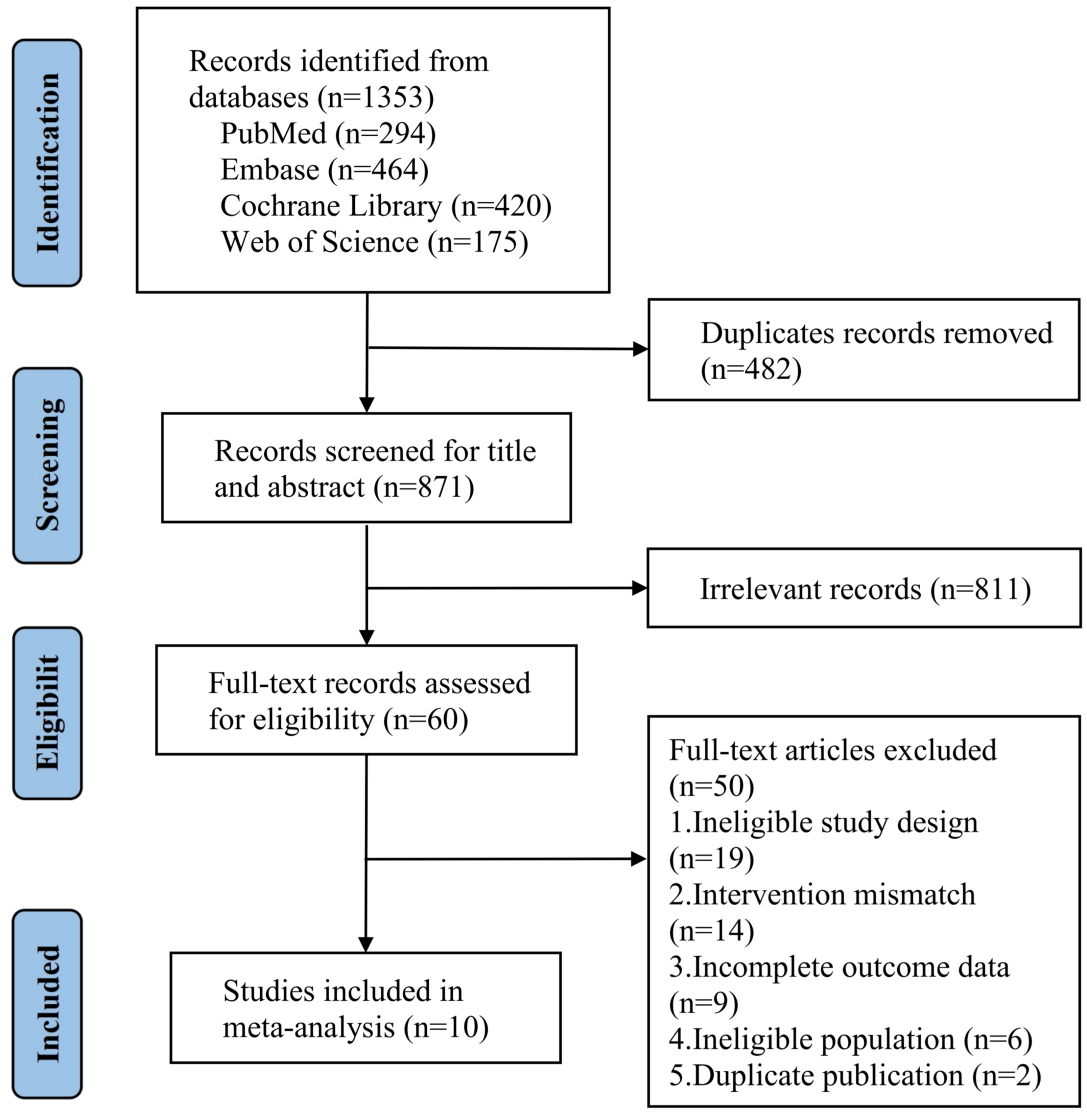
PRISMA flow diagram of the study selection process.

Figure 1 depicts the literature screening procedure in accordance with PRISMA recommendations. 1,353 pertinent records were found after a first thorough search of four databases, including PubMed, Embase, the Cochrane Library, and Web of Science. Following the removal of 482 duplicate entries, 811 irrelevant records were eliminated by screening the titles and abstracts of the remaining 871 records. The remaining 60 papers’ entire texts were next evaluated for eligibility, and 50 of them were eliminated because of ineligible study designs, mismatched interventions, incomplete outcome data, ineligible populations, and duplicate publications. In the end, 10 studies were included in this meta-analysis since they satisfied the predetermined inclusion criteria.

### 3.2 Eligible studies and characteristics

Table 1 lists the fundamental features of the 10 included studies. Every study was released between 2015 and 2024. Ten retrospective cohort studies (11–20) with 11,220 patients with locally advanced nasopharyngeal cancer made up the collection. All studies explicitly reported the use of intensity-modulated radiotherapy (IMRT) for the majority of patients, with the proportion of IMRT use reaching 100% in nine studies and 94.9% in one study. Regarding treatment regimens, three studies evaluated concurrent chemoradiotherapy (CCRT) alone, six studies evaluated induction chemotherapy (IC) followed by CCRT, and one study included cohorts treated with CCRT with or without targeted therapy. The threshold for distinguishing between high and low cumulative CCD was uniformly 200 mg/m^2^ across all included studies. The range of the median follow-up period was 28.2 to 67.5 months.

**Table 1:** Main characteristics of the included studies.

Author	Year	Country	Design	Sample size (n)		Age (year)	Sex (M/F)	Stage	Treatment	IMRT (%)	Median follow-up (month)	Outcomes
				CCD ≤ 200	CCD > 200							
Guo et al. (11)	2015	China	RE	392	99	43	359/132	II-IVb	CCRT	100	49	OS, DMFS
Lv et al. (12)	2018	China	RE	509	74	44	443/140	III-IVb	IC + CCRT	100	62	PFS
Liu et al. (13)	2019	China	RE	770	220	43	750/240	II-IV	IC + CCRT	100	28.2	PFS, DMFS
Gundog et al. (14)	2020	Turkey	RE	16	82	49.5	70/28	II-IVA	CCRT	94.9	41.5	OS, DMFS
Peng et al. (15)	2020	China	RE	2,754	706	—	—	II-IVA	IC + CCRT	100	44.7	OS, DMFS
Wen et al. (16)	2020	China	RE	291	291	44	451/131	III-IVA	IC + CCRT	100	53	OS, PFS, DMFS
Yang et al. (17)	2022	China	RE	2,938	185	44	2325/798	III-IVa	IC + CCRT	100	67.5	OS, PFS, DMFS
Jiang et al. (18)	2022	China	RE	111	111	43	191/31	III-IVa	IC + CCRT	100	46	OS, PFS, DMFS
Lin et al. (19)	2024	China	RE	714	182	43	677/219	III-IVa	IC + CCRT	100	62	OS, PFS, DMFS
Lan et al. (20)	2024	China	RE	380	395	45	563/212	II-IVa	CCRT±TT	100	51.6	PFS

RE indicates retrospective study, CCD indicates cumulative cisplatin dose (mg/m^2^); M indicates Male; F indicates Female; CCRT indicates Concurrent chemoradiotherapy; IC indicates induction chemotherapy, IMRT indicates intensity-modulated radiotherapy, OS indicates overall survival, — indicates not reported.

### 3.3 Risk of bias assessment

Table 2 summarizes the findings of the bias risk assessment for each study. For the 10 retrospective cohort studies, the NOS was used for assessment. A high-quality study was defined as having a score of at least six. The results showed that all studies had a score of ≥7, suggesting acceptable methodological quality. These 10 observational studies were all comparative studies with control groups. The detailed NOS scores are shown in Table 2. Seven of these investigations were given a score of nine, one an eight, and one a seven.

**Table 2:** Outcome data of included studies for meta-analysis.

Study	REP	SC	AE	NDO	COMP	AO	FU	AFU	Scoring
Guo et al. (11)	★	★	★	★	★★	★	★	★	9
Lv et al. (12)	★	★	★	★	★★	★	★	★	9
Liu et al. (13)	★	★	★	★	★★	★	★	★	9
Gundog et al. (14)	★	★	★	★	★	★	★	—	7
Peng et al. (15)	★	★	—	★	★★	★	★	★	8
Wen et al. (16)	★	★	★	★	★★	★	★	★	9
Yang et al. (17)	★	★	★	★	★★	★	★	★	9
Jiang et al. (18)	★	★	★	★	★★	★	★	★	9
Lin et al. (19)	★	★	★	★	★★	★	★	★	9
Lan et al. (20)	★	★	★	★	★★	★	★	★	9

EP indicates representativeness of the exposed cohort; SC indicates selection of the non-exposed cohort; AE indicates ascertainment of exposure; NDO indicates demonstration that outcome of interest was not present at start of study; COMP indicates comparability of cohorts; AO indicates assessment of outcome; FU indicates follow-up long enough for outcomes to occur; AFU indicates adequacy of follow-up completeness; — indicates not rated.

### 3.4 OS

**Figure 2:**
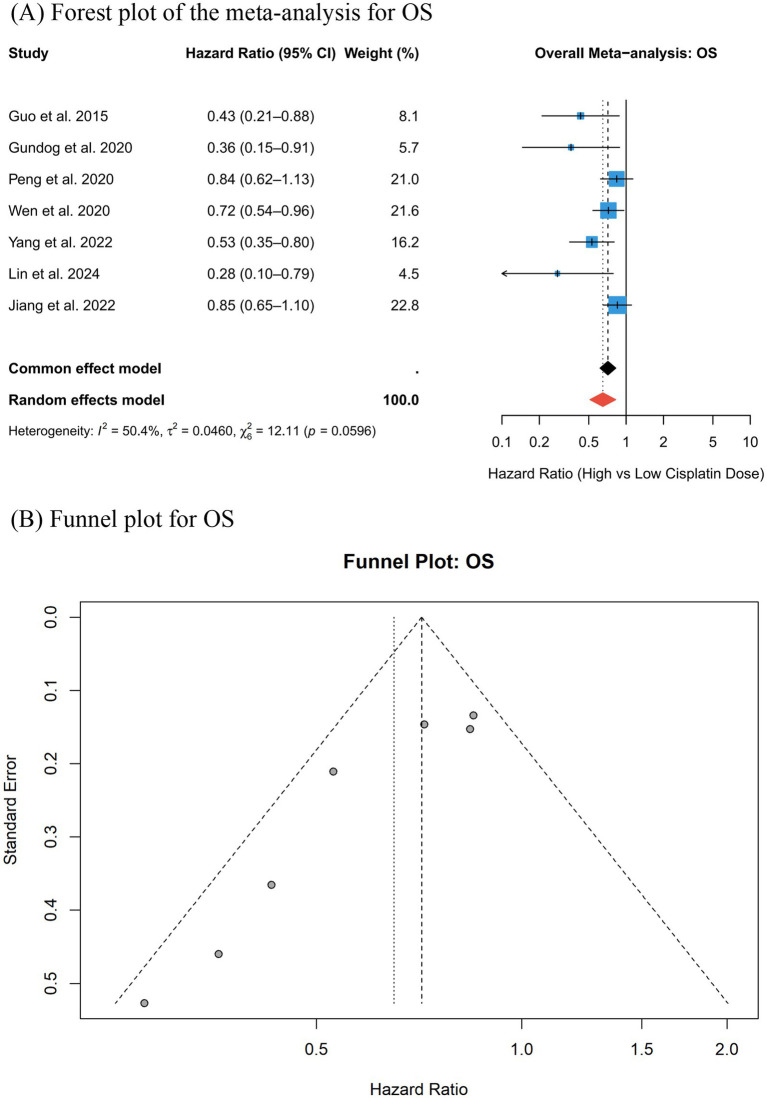
Comparison of OS between high-dose and low-dose cisplatin in locally advanced nasopharyngeal carcinoma. **(A)** Forest plot; **(B)** Funnel plot. OS indicates overall survival.

**Figure 3:**
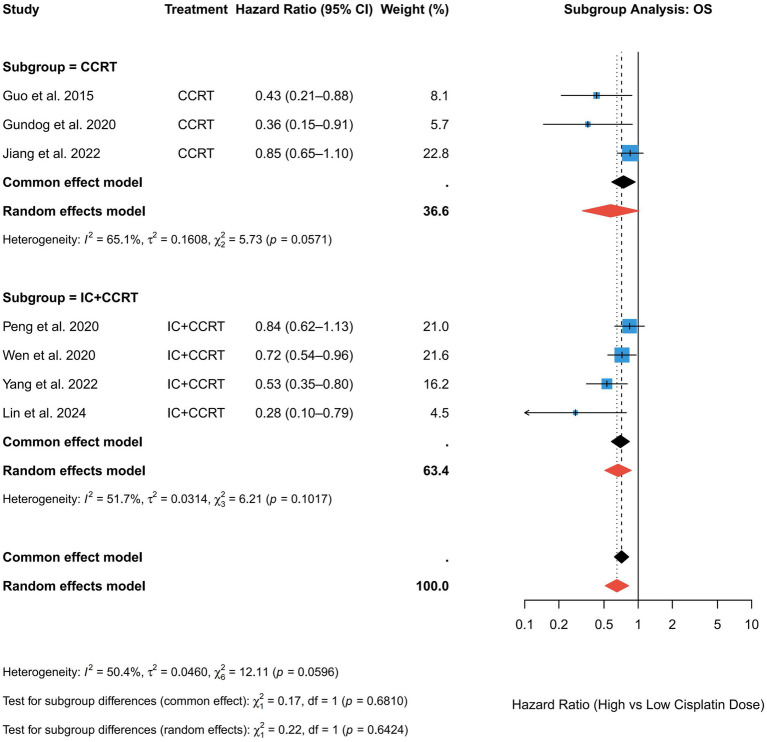
Subgroup analysis of OS by treatment modality. OS indicates overall survival.

**Figure 4:**
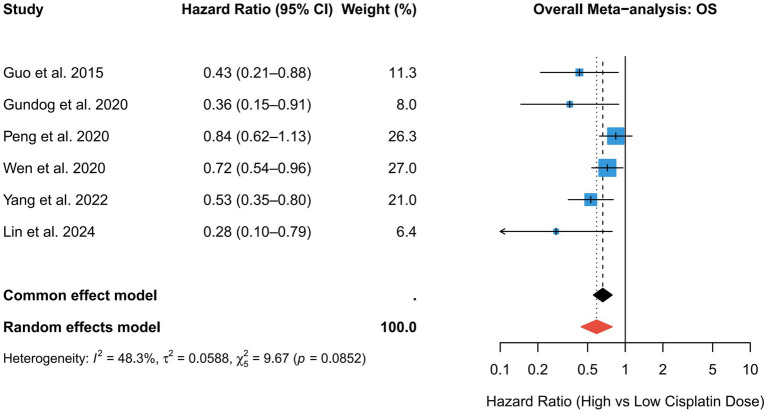
Forest plot of OS (after leave-one-out). OS indicates overall survival.

With an HR of 0.65 (95% CI: 0.51–0.82, *p* < 0.001) and moderate heterogeneity (I^2^ = 50.4%, *P* for heterogeneity = 0.060), the pooled results demonstrated that high-dose cisplatin (CCD > 200 mg/m^2^) was associated with a statistically significant improvement in OS compared with low-dose cisplatin (CCD ≤ 200 mg/m^2^; Figure 2A). Because the distribution of studies was approximately symmetric around the pooled effect, evaluation using a funnel plot revealed no clear evidence of publication bias (Figure 2B). Subgroup analysis by treatment modality was performed to explore the source of heterogeneity. In the CCRT subgroup, high-dose cisplatin showed a trend toward improved OS (HR = 0.57, 95% CI: 0.32–1.02, *p* = 0.056), with moderate-to-high heterogeneity (I^2^ = 65.1%, P for heterogeneity = 0.057). In the IC + CCRT subgroup, high-dose cisplatin was associated with a statistically significant improvement in OS (HR = 0.66, 95% CI: 0.51–0.87, *p* = 0.003), with moderate heterogeneity (I^2^ = 51.7%, *P* for heterogeneity = 0.102). However, the test for subgroup differences revealed no statistically significant interaction between the two treatment modalities (*P* for interaction = 0.642), indicating that treatment modality was not a significant source of heterogeneity (Figure 3). To confirm the stability of the OS results and investigate heterogeneity further, sensitivity analysis utilizing the leave-one-out method was carried performed. After excluding Jiang et al. (18), the pooled HR for OS was 0.59 (95% CI: 0.44–0.79), and the heterogeneity was reduced from 50.4 to 48.3% (Figure 4).

### 3.5 PFS

**Figure 5:**
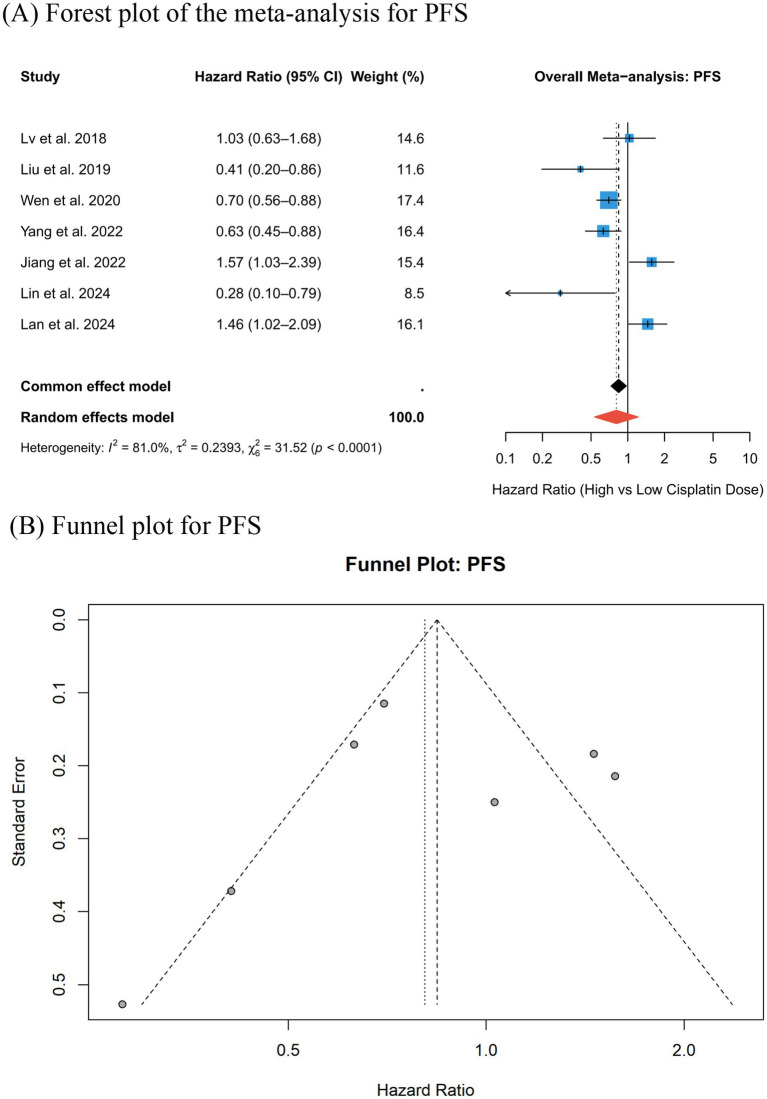
Comparison of PFS between high-dose and low-dose cisplatin in locally advanced nasopharyngeal carcinoma. **(A)** Forest plot; **(B)** Funnel plot. PFS indicates progression-free survival.

**Figure 6:**
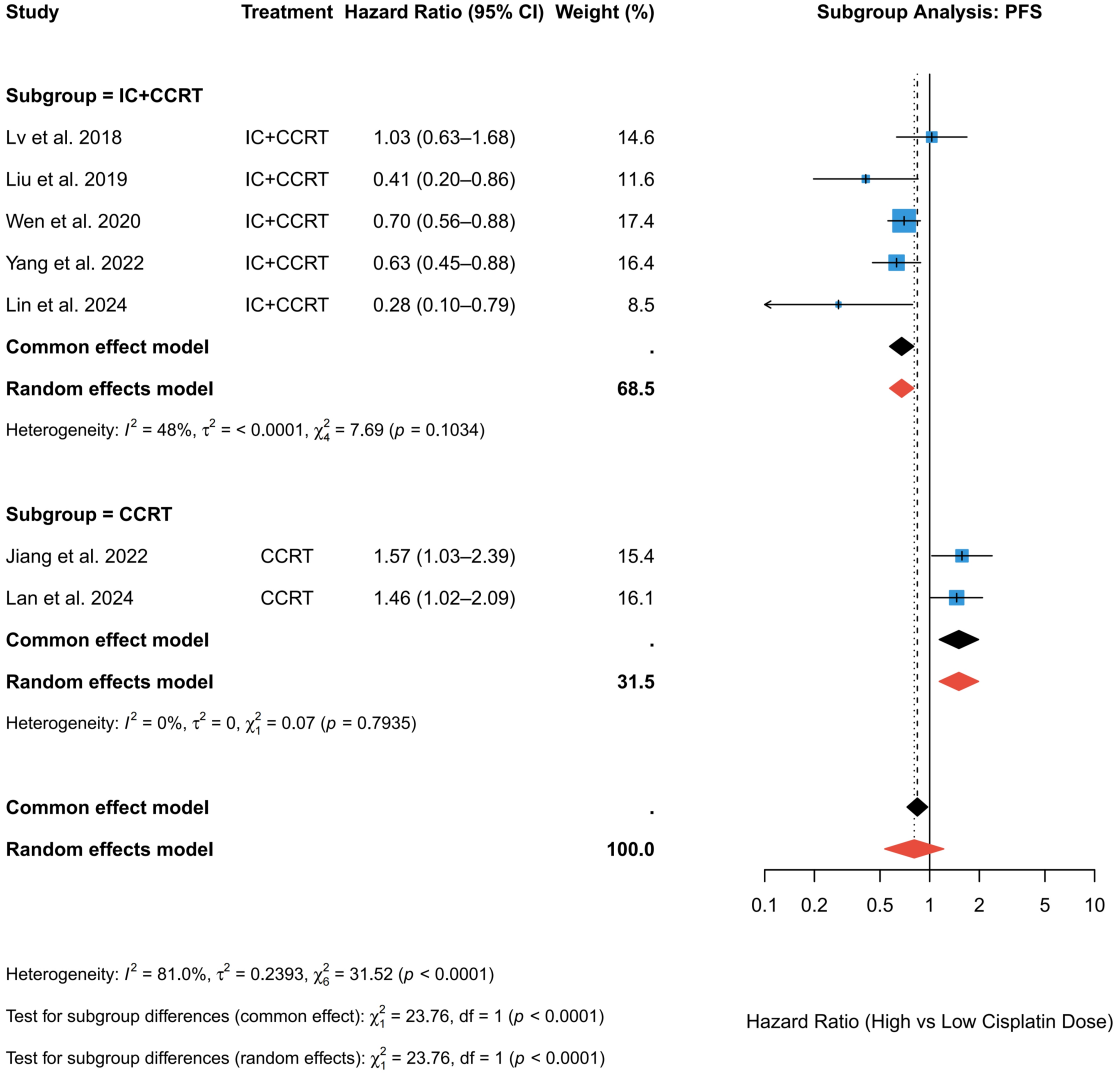
Subgroup analysis of PFS by treatment modality. PFS indicates progression-free survival.

With an HR of 0.81 (95% CI: 0.54–1.22, *p* = 0.305) and substantial heterogeneity (I^2^ = 81.0%, *P* for heterogeneity<0.001), the pooled findings revealed no statistically significant difference in PFS between the high-dose cisplatin group (CCD > 200 mg/m^2^) and the low-dose cisplatin group (CCD ≤ 200 mg/m^2^; Figure 5A). Because the distribution of studies was uneven around the pooled effect, evaluation using a funnel plot indicated possible publication bias (Figure 5B). Subgroup analysis by treatment modality was performed to explore the source of heterogeneity. In the IC + CCRT subgroup, high-dose cisplatin was associated with a statistically significant improvement in PFS (HR = 0.68, 95% CI: 0.57–0.80, *p* < 0.001), with moderate heterogeneity (I^2^ = 48.0%, *P* for heterogeneity = 0.103). In contrast, in the CCRT subgroup, high-dose cisplatin was associated with a statistically significant increase in PFS risk (HR = 1.51, 95% CI: 1.15–1.98, *p* = 0.003), with no heterogeneity (I^2^ = 0%, *P* for heterogeneity = 0.794). Treatment modality was the main cause of heterogeneity in the PFS analysis, according to the test for subgroup differences, which showed a highly statistically significant difference between the two treatment modalities (*p* < 0.001; Figure 6).

### 3.6 DMFS

**Figure 7:**
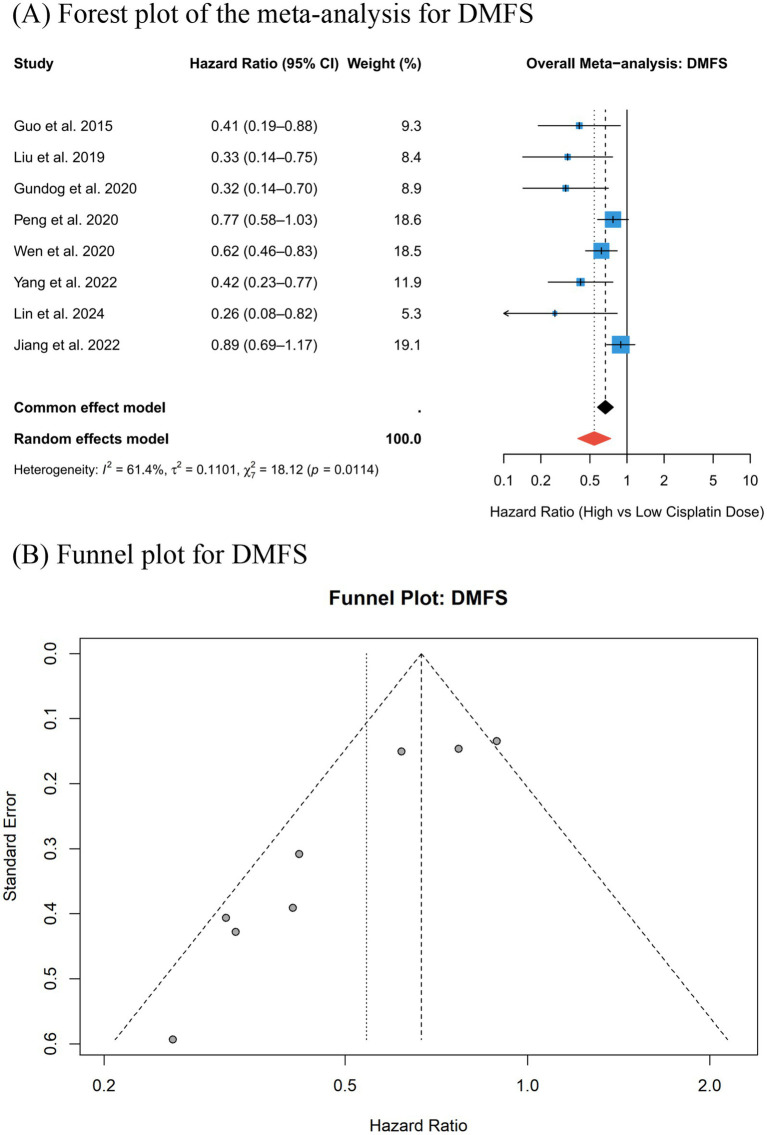
Comparison of DMFS between high-dose and low-dose cisplatin in locally advanced nasopharyngeal carcinoma. **(A)** Forest plot; **(B)** Funnel plot. DMFS indicates distant metastasis-free survival.

**Figure 8:**
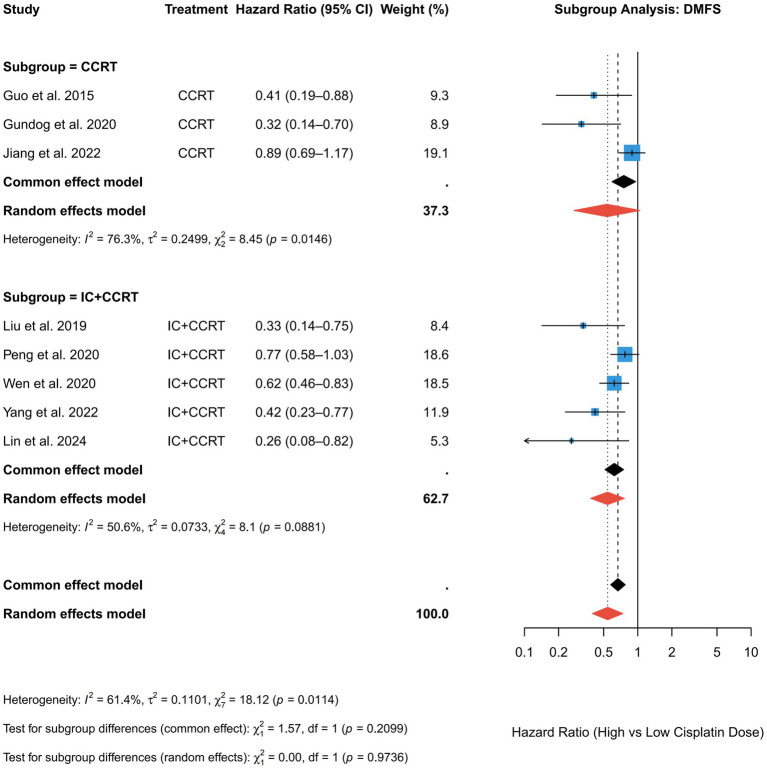
Subgroup analysis of distant metastasis-free survival by treatment modality. DMFS indicates distant metastasis-free survival.

**Figure 9:**
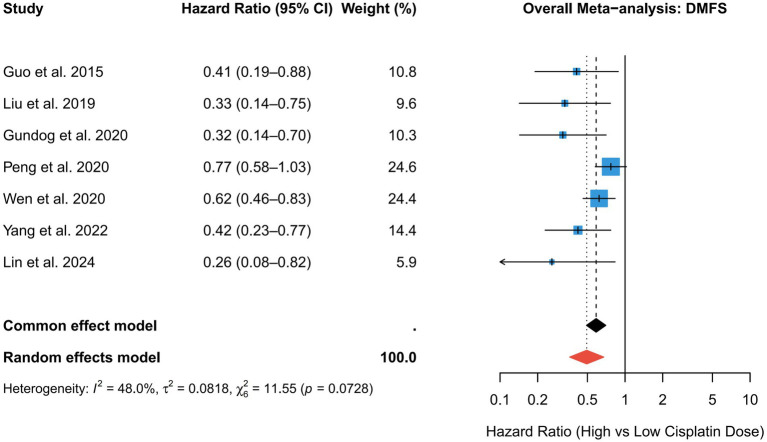
Forest plot of DMFS. DMFS indicates distant metastasis-free survival.

With an HR of 0.54 (95% CI: 0.40–0.74, *p* < 0.001) and moderate heterogeneity (I^2^ = 60.0%, *P* for heterogeneity = 0.011), the pooled results demonstrated that high-dose cisplatin (CCD > 200 mg/m^2^) was linked to a statistically significant improvement in DMFS when compared with low-dose cisplatin (CCD ≤ 200 mg/m^2^; Figure 7A). Because the distribution of studies was uneven around the pooled effect, a funnel plot assessment revealed possible publication bias (Figure 7B). Subgroup analysis by treatment modality was performed to explore the source of heterogeneity. In the CCRT subgroup, high-dose cisplatin showed a trend toward improved DMFS (HR = 0.54, 95% CI: 0.28–1.04, *p* = 0.066), with moderate-to-high heterogeneity (I^2^ = 76.3%, *P* for heterogeneity = 0.015). In the IC + CCRT subgroup, high-dose cisplatin was associated with a statistically significant improvement in DMFS (HR = 0.54, 95% CI: 0.38–0.76, *p* < 0.001), with moderate heterogeneity (I^2^ = 50.6%, *P* for heterogeneity = 0.088). Treatment modality was not a significant source of heterogeneity, according to the test for subgroup differences, which found no statistically significant difference between the two treatment modalities (*p* = 0.974; Figure 8). The stability of the DMFS results was confirmed through sensitivity analysis utilizing the leave-one-out method. After excluding Jiang et al. (18), the pooled HR for DMFS was 0.49 (95% CI: 0.36–0.68), and the heterogeneity was reduced from 61.4 to 48.0% (*P* for heterogeneity = 0.0728; Figure 9).

### 3.7 Toxicity analysis

Toxicity data were available from a subset of the included studies, with the number of studies contributing to each analysis ranging from 2 to 7. The pooled results for acute and late AEs are summarized in Table 3. The incidence of grade 3–4 leukopenia (RR = 0.84, 95% CI: 0.55–1.28, *p* = 0.426), grade 3–4 ALT elevation (RR = 0.98, 95% CI: 0.27–3.60, *p* = 0.980), any-grade anemia (RR = 0.96, 95% CI: 0.91–1.01, *p* = 0.093), thrombocytopenia (RR = 0.89, 95% CI: 0.68–1.18, *p* = 0.427), and neutropenia (Grade 1–4: RR = 0.90, 95% CI: 0.80–1.02, *p* = 0.099; Grade 3–4: RR = 1.07, 95% CI: 0.83–1.37, *p* = 0.600). The incidence of any-grade leukopenia (RR = 0.91, 95% CI: 0.86–0.97, *p* = 0.002), any-grade ALT elevation (RR = 0.69, 95% CI: 0.58–0.82, *p* < 0.001), and creatinine elevation at both any-grade (RR = 0.73, 95% CI: 0.60–0.88, *p* = 0.001) and grade 3–4 (RR = 0.20, 95% CI: 0.04–0.89, *p* = 0.035) were significantly lower in the CCD ≤ 200. The rates of grade 3–4 hearing impairment (RR = 0.60, 95% CI: 0.33–1.08, *p* = 0.089), any-grade xerostomia (RR = 0.94, 95% CI: 0.85–1.03, *p* = 0.183), trismus (RR = 1.05, 95% CI: 0.77–1.44, *p* = 0.741), and cranial neuropathy (RR = 0.87, 95% CI: 0.65–1.15, *p* = 0.324) were not significantly different between the two groups. However, the incidence of any-grade hearing impairment (RR = 0.81, 95% CI: 0.70–0.94, *p* = 0.005), any-grade skin fibrosis (RR = 0.77, 95% CI: 0.64–0.92, *p* = 0.004), and grade 3–4 skin fibrosis (RR = 0.43, 95% CI: 0.21–0.89, *p* = 0.023) was considerably lower in the lower-dose group.

**Table 3:** Summary of adverse events.

Adverse events	No. of studies	Sample size (n)		RR (95% CI)	*p*
		CCD ≤ 200 mg/m^2^	CCD > 200 mg/m^2^		
Acute toxicity					
Anemia (Grade 1–4)	7	6,281	1941	0.96 (0.91–1.01)	0.093
Anemia (Grade 3–4)	7	6,281	1941	0.71 (0.39–1.29)	0.258
Thrombocytopenia (Grade 1–4)	7	6,281	1941	0.89 (0.68–1.18)	0.427
Thrombocytopenia (Grade 3–4)	7	6,281	1941	1.02 (0.63–1.63)	0.949
Leukopenia (Grade 1–4)	7	6,281	1941	0.91 (0.86–0.97)	0.002
Leukopenia (Grade 3–4)	7	6,281	1941	0.84 (0.55–1.28)	0.426
Neutropenia (Grade 1–4)	7	6,281	1941	0.90 (0.80–1.02)	0.099
Neutropenia (Grade 3–4)	7	6,281	1941	1.07 (0.83–1.37)	0.600
AST Elevation (Grade 1–4)	5	4,332	1,201	0.79 (0.59–1.04)	0.091
AST Elevation (Grade 3–4)	5	4,332	1,201	0.27 (0.04–1.91)	0.189
ALT Elevation (Grade 1–4)	5	4,332	1,201	0.69 (0.58–0.82)	<0.001
ALT Elevation (Grade 3–4)	5	4,332	1,201	0.98 (0.27–3.60)	0.980
Creatinine Elevation (Grade 1–4)	3	6,870	1,376	0.73 (0.60–0.88)	0.001
Creatinine Elevation (Grade 3–4)	3	6,870	1,376	0.20 (0.04–0.89)	0.035
Late toxicity					
Hearing Impairment (Grade 1–4)	4	4,619	880	0.81 (0.70–0.94)	0.005
Hearing Impairment (Grade 3–4)	4	4,619	880	0.60 (0.33–1.08)	0.089
Xerostomia (Grade 1–4)	3	4,508	769	0.94 (0.85–1.03)	0.183
Xerostomia (Grade 3–4)	3	4,508	769	1.24 (0.60–2.55)	0.561
Trismus (Grade 1–4)	2	4,116	670	1.05 (0.77–1.44)	0.741
Trismus (Grade 3–4)	2	4,116	670	1.94 (0.64–5.89)	0.243
Cranial Neuropathy (Grade 1–4)	2	4,116	670	0.87 (0.65–1.15)	0.324
Cranial Neuropathy (Grade 3–4)	2	4,116	670	0.50 (0.21–1.18)	0.114
Skin Fibrosis (Grade 1–4)	3	4,508	769	0.77 (0.64–0.92)	0.004
Skin Fibrosis (Grade 3–4)	3	4,508	769	0.43 (0.21–0.89)	0.023

RR indicates relative risk, CI indicates confidence interval.

## 4 Discussion

This systematic review and meta-analysis evaluated the efficacy differences of various CCD in the treatment of LA-NPC in the IMRT era, providing an updated synthesis of the available evidence. A greater CCD (≥200 mg/m^2^) is linked to considerably better DMFS (HR = 0.54, 95% CI: 0.40–0.74, *p* < 0.001), according to the core analysis, while the benefit for overall survival showed a favorable trend approaching statistical significance (HR = 0.65, 95% CI: 0.51–0.82, *p* < 0.001) with moderate heterogeneity (I^2^ = 50.4%). Both conclusions exhibited a high degree of robustness in sensitivity analyses. However, the analysis for PFS did not yield a definitive conclusion 0.81 (95% CI: 0.54–1.22, *p* = 0.305), showing substantial heterogeneity (I^2^ = 81.0%) and fragility, indicating that current evidence on PFS remains inconsistent. These findings indicate that the association between cisplatin dose and clinical outcomes in LA-NPC is endpoint-specific, with DMFS being the most consistent endpoint responsive to higher cisplatin doses in the IMRT era.

Our findings of a DMFS benefit associated with higher CCD are consistent with the dose intensity principle, which posits that achieving sufficient cytotoxic drug exposure is fundamental for eradicating tumor cells, particularly micrometastases (21). However, the significant systemic toxicity of cisplatin poses a major challenge in its clinical application and is a key driver for exploring novel delivery methods to reduce toxicity (22). In this study, the benefit conclusion is predicated on patients being able to tolerate and complete the prescribed treatment. Within the modern IMRT technological framework, the precision and optimizability of its planning and delivery have been continuously validated and enhanced through advanced dose prediction models and automated planning functions, signifying that IMRT can provide more excellent and consistent local control than before (23, 24). Consequently, our results suggest that, with local control robustly assured, the core value of adequate cisplatin may lie more in its systemic therapeutic effect. This is corroborated by key studies included in our analysis. For instance, research by Wen et al. (16) demonstrated an OS benefit for the high-dose group in the high-risk subgroup (HR = 0.72), and Yang et al. (17) also showed a survival advantage (HR = 0.53). This indicates that a major contribution of concurrent cisplatin may be the clearance of circulating tumor cells and subclinical distant metastases, thereby translating the local control advantage of IMRT into DMFS and ultimately OS benefits, which are critical for long-term survival. This finding suggests that, under the condition that local control is reliably guaranteed in the IMRT era, the role of concurrent cisplatin chemotherapy may extend beyond the traditional role of radiosensitization and partially lean toward systemic consolidation for patients at high risk of distant metastasis of nasopharyngeal carcinoma. This provides a preliminary research basis for exploring the optimization of cisplatin dosage based on the goal of controlling distant metastasis in selected patient groups.

In contrast to the relatively consistent signal for DMFS, the analysis for PFS exhibited high heterogeneity (I^2^ = 81.0%) and fragile conclusions, highlighting the clinical complexity in assessing the cisplatin dose effect. Our study observed a key paradox: the overall PFS benefit was not statistically significant, yet subgroup analysis revealed a significant divergence based on treatment modality. In the IC + CCRT subgroup, higher CCD was associated with significantly improved PFS (HR = 0.68, 95% CI: 0.57–0.80), whereas in the CCRT alone subgroup, higher CCD paradoxically appeared harmful (HR = 1.51, 95% CI: 1.15–1.98), with a statistically significant subgroup difference (*p* < 0.001). This striking discrepancy may explain the overall null finding and high heterogeneity, and this observation is consistent with contemporary NPC dose-effect research that adopted a unified cisplatin dose cut-off for interstudy comparability (11, 12, 19). This divergence may be attributed to the distinct clinical implications of cisplatin dose in different treatment modalities, cisplatin serves as a consolidation agent after induction response in IC + CCRT, while it simultaneously acts as a radiosensitizer and systemic therapy in CCRT alone. This inconsistency suggests that the effect of CCD on PFS may be influenced by multiple factors, including variation in patient tolerance and cisplatin suitability, as well as differences in platinum agents used across studies (25, 26). However, given the exploratory nature of subgroup analyses and the contradictory directions of effect, these findings should be interpreted with caution and do not support a causal conclusion. Sensitivity analysis confirmed that the pooled PFS result was heavily influenced by individual studies. For instance, Lan et al. (20) (HR = 1.46) suggested potential harm from higher doses, aligning with the CCRT subgroup trend.

More importantly, the value and interpretation of PFS itself as an efficacy endpoint are inherently controversial. For example, in drug reimbursement decisions, health technology assessment agencies in different countries may hold fundamentally divergent views on treatment regimens offering only marginal PFS benefit without clear OS benefit (27). This macro-level controversy is also reflected in clinical research data from other cancers. For example, it is sometimes questioned whether PFS is a reliable surrogate endpoint for predicting long-term survival in systemic treatments such as immunotherapy for non-small cell lung cancer (28). Consequently, in this study, PFS may suffer from a low signal to noise ratio due to interference from various non-dose-related clinical and methodological factors, coupled with uncertainty in reflecting the ultimate patient benefit. Its substantial heterogeneity is a true reflection of the complexity of clinical practice and the plurality of interpretations of the efficacy endpoint itself. This finding strongly suggests that future research on cisplatin dose optimization in NPC should place greater emphasis on and prioritize reporting more clinically definitive hard endpoints like DMFS and OS.

Subgroup analysis provided exploratory insights that may inform individualized treatment. In both OS and DMFS analyses, we observed a clinically meaningful trend: the benefit of high-dose cisplatin was more pronounced and consistent in the IC + CCRT subgroup, whereas it was attenuated and less stable in the CCRT alone subgroup. For OS, the IC + CCRT subgroup showed a statistically significant improvement (HR = 0.66, 95% CI: 0.51–0.87, *p* = 0.003), while the CCRT subgroup showed a non-significant trend (HR = 0.57, 95% CI: 0.32–1.02, *p* = 0.056), with no significant interaction (*p* = 0.642). For DMFS, the CCRT subgroup showed a non-significant trend toward improvement (HR = 0.54, 95% CI: 0.28–1.04, *p* = 0.066), while the IC + CCRT subgroup demonstrated a statistically significant improvement (HR = 0.54, 95% CI: 0.38–0.76, *p* < 0.001), with no significant interaction between subgroups (*p* = 0.974). These findings suggest that while the dose effect may be present across treatment modalities, the magnitude is amplified in patients receiving induction chemotherapy. Patients who have received induction chemotherapy may themselves represent a selected group. Studies show that comprehensive models based on indicators like Epstein–Barr virus DNA and clinical stage can effectively identify patients most likely to benefit from induction chemotherapy (29). For these patients, deep response after induction chemotherapy may indicate that their tumors are relatively chemotherapy-sensitive. In such cases, using adequate cisplatin during the concurrent phase for consolidation and intensification may be decisive for eradicating residual sensitive clones and preventing distant metastasis. This concept aligns with findings from Liu et al. (13), who demonstrated that the benefit of high-dose cisplatin was concentrated in patients achieving response to induction chemotherapy. Further research indicates that post-induction chemotherapy Epstein–Barr virus DNA levels retain significant prognostic value, suggesting that even after induction chemotherapy, the treatment response can still guide the intensity of subsequent therapy, supporting the strategy of intensifying concurrent treatment for good responders (30). These exploratory insights may inform individualized treatment decisions but require rigorous prospective validation. Given the post-hoc nature of subgroup analyses, these results should be considered hypothesis-generating rather than practice-changing.

Conversely, in patients receiving CCRT alone, the initial tumor burden is large and heterogeneous. Their prognosis may depend more comprehensively on the quality of radiotherapy itself, the intrinsic aggressive biological characteristics of the tumor, and whether effective systemic treatment was promptly employed (31). The lack of uniform benefit from simply increasing cisplatin dose in this group in our study, with OS showing no significant difference and PFS even suggesting potential harm, suggests that tumors in some CCRT patients may harbor intrinsic or early cisplatin resistance. Therefore, future treatment strategies for this population may need to move beyond mere dose intensification and instead explore personalized biomarker-based risk-adapted strategies. For high-risk patients, such as those with elevated pretreatment Epstein–Barr virus DNA levels, consideration could be given to more potent systemic therapy combinations including combined immunotherapy, which is emerging as a promising approach in LA-NPC treatment. For patients with specific molecular subtypes, exploring combination with targeted agents against resistance pathways like autophagy or DNA damage repair may help reverse or overcome resistance.

Our toxicity analysis revealed that while most acute adverse events were comparable between dose groups, the lower-dose cohort (CCD ≤ 200 mg/m^2^) demonstrated significantly reduced risks of certain toxicities, including any-grade leukopenia (*p* = 0.002), ALT elevation (*p* < 0.001), and creatinine elevation (*p* = 0.001 for any-grade, *p* = 0.035 for grade 3–4). For late toxicities, the lower-dose group showed significantly lower incidences of any-grade hearing impairment (*p* = 0.005) and skin fibrosis (*p* = 0.004 for any-grade, *p* = 0.023 for grade 3–4). These findings align with the well-established systemic toxicity profile of cisplatin, which involves oxidative stress, inflammatory responses, and apoptotic cell death in normal tissues (32). Specifically, cisplatin-induced nephrotoxicity is mediated through renal tubular cell injury via reactive oxygen species generation and activation of the renin-angiotensin system (33), while ototoxicity results from accumulation of cisplatin in cochlear hair cells, leading to mitochondrial dysfunction and programmed cell death (34). The reduced toxicity burden associated with lower cumulative doses supports the clinical rationale for individualized dose optimization, particularly in patients at higher risk of treatment-related complications.

In recent years, immune checkpoint inhibitors have made significant progress in the treatment of LA-NPC. RCTs by Liu et al. (35) and Liang et al. (36) demonstrated that combining immunotherapy with chemoradiotherapy, whether in neoadjuvant+adjuvant or adjuvant mode, significantly improved survival benefits in patients with LA-NPC. Wang et al.’s (37) phase II study also showed that camrelizumab combined with chemoradiotherapy had good efficacy and safety in non-endemic regions. These studies indicate that the integration of immunotherapy is profoundly changing the traditional treatment landscape of LA-NPC and offering a new clinical perspective for cisplatin dose strategies in the IMRT era. This study focuses on the traditional cisplatin-based CCRT in the IMRT era, analyzing the association between different cumulative cisplatin doses and survival outcomes as well as toxicities. Although we did not directly include immunotherapy-related regimens, our findings can still serve as a reference baseline for exploring the optimal cisplatin dose in combination with immunotherapy. In the immunotherapy era, whether the cisplatin dose strategy needs to be adjusted, and whether the dose can be appropriately reduced while ensuring anti-tumor efficacy to lower toxicity, requires individualized assessment based on treatment regimens and remains a key clinical question. Future large-sample prospective RCTs are needed to explore the optimal cisplatin dose in combined immunotherapy and chemoradiotherapy under different treatment modalities, providing high-level evidence for individualized dose strategies.

This study has the following limitations. First, the included studies were predominantly retrospective in design, carrying residual confounding bias that cannot be fully eliminated despite the use of multivariate adjustments and propensity score matching in some studies. Second, the distinct clinical implications of cisplatin dose across different treatment modalities may introduce inherent heterogeneity, limiting the direct extrapolation of our findings to clinical practice. Third, the OS benefit did not reach statistical significance, which may reflect limited statistical power despite the large sample size. Fourth, systematic toxicity data were lacking, precluding a formal risk–benefit assessment of high-dose regimens. Fifth, all conclusions are based predominantly on Chinese populations, with only one study from Turkey, which limits the generalizability of our findings to other ethnic groups and healthcare settings. Future research should focus on conducting prospective RCTs to directly validate individualized dose strategies, systematically evaluating the association between dose intensity and toxicity profiles, and exploring biomarkers predictive of dose sensitivity. The ultimate goal is to achieve a paradigm shift from empirical full-dose chemotherapy to evidence-based precision dose management.

## 5 Conclusion

A higher CCD was associated with improved DMFS in patients with LA-NPC in the IMRT era, particularly in those who received induction chemotherapy. However, the effect on PFS was inconsistent and highly heterogeneous, and the benefit for OS did not reach statistical significance. These findings indicate that treatment modality may modulate the response to higher cisplatin doses, and do not support a broadly generalizable dose threshold. Clinical decision-making should integrate treatment modality, patient tolerance, and individual risk stratification with a balance of efficacy and toxicity. Future prospective studies are needed to validate risk-adapted and modality-specific dosing strategies.

## Data Availability

The original contributions presented in the study are included in the article/supplementary material, further inquiries can be directed to the corresponding author.
